# Spectrophotometric Determination of Certain Benzimidazole Proton Pump Inhibitors

**DOI:** 10.4103/0250-474X.44605

**Published:** 2008

**Authors:** A. A. Syed, Ayesha Syeda

**Affiliations:** Department of Studies in Chemistry, University of Mysore, Mysore-570 006, India

**Keywords:** Benzimidazole, prussian blue, proton pump inhibitors, spectrophotometry

## Abstract

Spectrophotometric method for the determination of certain proton pump inhibitors belonging to the benzimidazole class of compounds has been developed. The method is based on the reaction of omeprazole, lansoprazole, pantoprazole, rabeprazole and esomeprazole with iron (III) and subsequent reaction with ferricyanide under neutral condition which yields Prussian blue product with maximum absorption at 720–730 nm. The commonly encountered excipients and additives that often accompany pharmaceutical preparations did not interfere with the determination. The method was applied for the determination of omeprazole, lansoprazole, pantoprazole, rabeprazole and esomeprazole in pharmaceutical preparations and no difference was found statistically. Thus, the spectrophotometric method can be applied as inexpensive, rapid, easy, accurate and precise method for the routine analysis of the five proton pump inhibitors in pharmaceutical preparations.

Omeprazole (OMZ), lansoprazole (LNZ), pantoprazole (PNZ), rabeprazole (RBZ) and esomeprazole (EMZ) belong to a class of antisecretory compounds. These compounds are acid labile and reversibly transformed in acidic medium to a sulfonamide^1^. They are referred to as proton pump inhibitors (PPIs) and were introduced for the management of duodenal ulcer, gastric ulcer or pathogenic hypersecretory condition^2^. Gastric PPIs are prodrugs that require an acid- induced activation. It is a weak base that is converted to its active form by gastric acid before acting on the proton pump. It inhibits gastric acid secretion by covalently binding to the proton pump (H^+^/K^+^ATPase)^3^.

Development of new methods capable of determining drug concentrations in pharmaceutical formulations is important. Contemporary analytical methodologies adopted to determine antiulcer drugs in different matrices include electroanalytical techniques^4^–^8^, chromatographic methods 9–14 and automated methods such as flow injection analysis^15^,^16^. However, these methods are deficient with respect to specificity, sensitivity, simplicity and short time analysis.

A survey of literature revealed that no data has been published on the UV-visible spectrophotometric methods for RBZ and EMZ and very limited methods are available for the spectrophotometric determination of OMZ, LNZ and PNZ in formulations. The details of existing spectrophotometric reagents and their spectral characteristics are enumerated in Table 1.

**TABLE 1:** COMPARISON OF VISIBLE SPECTROPHOTOMETRIC METHODS FOR THE DETERMINATION OF ANTIULCER DRUGS

Reagent	Coloured species	Drugs analyzed	λmax nm	Range μg ml-1	Remarks	Ref
Iron(III)	radical cation	PNZ	455	30–300	Heating at 60° for 30 min	17
Iron(III)	radical cation	OMZ	411	15–95	Heating at 65±5° for 15 min	17
Chromium(III)	radical cation	OMZ	339	10–60	Heating at 65±5° for 10 min	17
Cobalt(II)	radical cation	OMZ	523	15–150	Heating at 65±5° for 35 min	17
2,3-dichloro-5, 6-dicyano-1, 4-benzoquinone	charge transfer complex	LNZ, PNZ	457	10–90, 10-60	Reaction carried out in acetonitrile medium	18
Iodine	charge transfer complex	LNZ, PNZ	293, 359	1.5.6.7, 17.7.141.6	Reaction carried out in chloroform medium	18
Eosin and copper(II)	molecular complex	LNZ, PNZ	549	3.7.16.6, 4.3.25.9	Heating at 60° for 20 min, heating at 70° for 25 min	18
MBTH	coupled product	OMZ	660	1.0.10.0	Stable only for 20 min	19
*m*-aminophenol	coupled product	OMZ	420	2.0–32	Stable only for 10 min	19
Proposed method	complex	OMZ, LNZ, PNZ, RBZ, EMZ	720–730	0.2-4.0	Reaction carried out at room temperature and stable for 3 h	-

OMZ is omeprazole; LNZ is lansoprazole; PNZ is pantoprazole; RBZ is rabeprazole; EMZ is esomeprazole, Ref denotes reference

An attempt was made to develop simple, sensitive and selective spectrophotometric procedure for the determination of OMZ, LNZ, PNZ, RBZ and EMZ in both preformulations and dosage forms. The proposed procedure involves the reduction of iron(III) to iron(II) which subsequently reacts with ferricyanide to form a Prussian blue product in neutral medium having maximum absorption at 720–730 nm. This method has distinct advantages of sensitivity and stability and also does not require heating or distillation and exhibits reliability due to reproducibility.

UV/Vis spectrophotometer Uvidec-610 type with 1.0-cm matched cell (Jasco, Tokyo, Japan) was employed for measuring the absorbance values. Omeprazole, lansoprazole, pantoprazole, rabeprazole from Cipla, India and commercial tablets of esomeprazole, ferric chloride and potassium ferricyanide (BDH, India) were used. All other chemicals and solvents used were of analytical grade. Double distilled water was used throughout. Samples of the drugs (100 mg) were dissolved in about 10.0 ml alcohol and made up to 100-ml in a volumetric flask with distilled water and stored in a refrigerator. Standard solutions of the drugs were prepared every day by diluting the stock solution with distilled water. Aqueous solution of 0.0025 M ferric chloride containing few drops of 5 M hydrochloric acid and 0.001 M potassium ferricyanide was prepared in double distilled water.

Aliquots of standard solutions of OMZ (2.0–70.0 μg), LNZ (5.0–90.0 μg), PNZ (5.0–100.0 μg), RBZ (5.0–80.0 μg) and EMZ (5.0–80.0 μg) were transferred into 25–ml calibrated flask. Ferric chloride and potassium ferricyanide each 3.0 ml were added to each flask, the contents were mixed well and kept aside for 20 min at 27°. It was diluted to the mark with distilled water. The absorbance was measured at 720–730 nm against the corresponding reagent blank and calibration graphs were constructed. The optical characteristics of the chromogen are presented in Table 2.

**TABLE 2:** OPTICAL CHARACTERISTICS OF THE CHROMOGEN USING IRON(III) IN THE PRESENCE OF FERRICYANIDE

	OMZ	LNZ	PNZ	RBZ	EMZ
Colour	Blue	Blue	Blue	Blue	Blue
λ_max_ (nm)	720	730	720	720	725
Stability (h)	3.0	2.5	3.0	2.5	3.0
Beer’s law (ng/ml)	80–2800	200–3600	200–4000	200–3200	200–3200
Molar absorptivity (L mol^-1^cm^-1^)	8.76×10^4^	6.78×10^4^	6.74×10^4^	6.44×10^4^	7.22×10^4^
Sandell’s sensitivity (μg cm^-2^)	0.004	0.005	0.006	0.005	0.005
Regression equation*				
Slope (a)	0.1702	0.1388	0.1069	0.1201	0.1120
Intercept (b)	0.0523	0.0411	0.0691	-0.0610	0.0141
Correlation coefficient	0.9853	0.9925	0.9844	0.9921	0.9899

* y= ax+b where x is the concentration of OMZ, LNZ, PNZ, RBZ or EMZ in μg/ml, OMZ is omeprazole; LNZ is lansoprazole; PNZ is pantoprazole; RBZ is rabeprazole; EMZ is esomeprazole

Twenty capsules each of OMZ and LNZ were emptied carefully and the mass of the collected contents was determined. The capsule contents were finely powdered in a mortar. In case of PNZ, RBZ and EMZ twenty tablets each were finely powdered. An accurately weighed 50 mg of the powdered drug was dissolved in about 10.0 ml of alcohol and filtered through a Whatman No. 42 filter paper. The filtrate was made up to 100-ml with distilled water in a volumetric flask. A suitable volume of the filtrate was accurately diluted with water to obtain a sample concentration of 10 μg/ml. An aliquot of this solution was treated as per the procedure described earlier for the determination of antiulcer drugs.

The method for determination of antiulcer drugs involves the reaction of the drugs with ferric salt, in the presence of potassium ferricyanide under neutral condition, to produce a Prussian blue coloured product with maximum absorption at 720–730 nm. The reaction involves the reduction of iron(III) by omeprazole, lansoprazole, pantoprazole, rabeprazole and esomeprazole to form iron(II), which subsequently reacts with ferricyanide to give a Prussian blue (PB) product in neutral medium.

The factors affecting the colour development such as reproducibility, sensitivity and adherence to Beer’s law were investigated using omeprazole as a model compound, since the other antiulcer drugs behaved similar to it.

A blue coloured product with maximum absorption at 720 nm was formed when omeprazole was allowed to react with ferric chloride, in presence of potassium ferricyanide in neutral medium. It was found that a 0.0025 M solution of ferric chloride in the range of 2.0-5.0 ml and 0.001 M solution of potassium ferricyanide in the range of 1.0-4.0 ml were necessary to achieve maximum colour intensity and stability of the blue colour. Hence, 3.0 ml each of ferric chloride and ferricyanide solutions were recommended.

The order of addition of ferric chloride, ferricyanide and drug solution for the formation of the blue complex was studied. No appreciable change in the absorbance or colour of the product was observed when the order of addition of these reactants was altered.

Table 2 shows the linear calibration ranges and equation parameters for this procedure. Separate determinations at different concentrations of each drug gave a coefficient of variation not exceeding 2%. The resultant product of the proposed method was studied at different temperatures. The coloured product was stable up to 3 h at room temperature.

Various additives and excipients that often accompany antiulcer drugs in pharmaceutical preparations such as lactose, glucose, starch, gum acacia, magnesium stearate and talc did not interfere, while vitamin C was found to interfere significantly.

The applicability of the method to assay pharmaceutical preparations was examined. Commercial capsules/tablets containing OMZ, LNZ, PNZ, RBZ and EMZ were successfully analyzed by the proposed method. The results obtained are listed in Table 3. The results were compared statistically using F- and t- tests. The calculated F- and t- values did not exceed the theoretical values. Therefore, it is concluded that there is no significant difference in the proposed method of analysis with respect to repeatability (F-test) and accuracy (t-test).

**TABLE 3:** DETERMINATION OF CERTAIN PROTON PUMP INHIBITORS IN COMMERCIAL SAMPLES BY THE PROPOSED METHOD

Drug	Label claim (mg per drug)	*Recovery% ±SD**	Additional analyte added (mg)	*Recovery% ±SD**	Reported method found%
Omelac capsule (Omeprazole)	20	98.1±1.11 (n=5)	20	100.2±0.84	97.2±1.09 [17] (n=5)
Lanpro capsule (Lansoprazole)	15	99.8±0.25 (n=5)	15	99.8±0.60	99.63±0.11 [18] (n=5)
Pan tablet (Pantoprazole)	20	99.0±1.20 (n=4)	20	99.0±1.02	98.5±0.61 [17] (n=4)
Rabeloc tablet (Rabeprazole)	20	99.0±0.72 (n=7)	20	100.8±0.72	98.4±0.44 [20] (n=7)
Raciper tablet (Esomeprazole)	20	99.1±0.92	20	99.4±0.92	-

* Proposed method

** standard deviation

The procedure described here is simple, rapid, sensitive, selective and cost effective. It is evident from the results that the recommended procedure is well suited for the assay and evaluation of drugs, in preformulation and dosage forms. It can be applied for direct determination of proton pump inhibitors in drug control laboratories.
